# Developmental outcomes of small infants at a high-risk clinic: A short-term longitudinal study

**DOI:** 10.4102/sajcd.v72i1.1099

**Published:** 2025-08-14

**Authors:** Tayla-Ann Macaskill, Maria du Toit, Renata Eccles, Marien A. Graham, Jeannie van der Linde

**Affiliations:** 1 Department of Speech-Language Pathology and Audiology, Faculty of Humanities, University of Pretoria, Pretoria, South Africa; 2 Department of Mathematics Education, College of Education, University of South Africa, Pretoria, South Africa

**Keywords:** small infants, developmental screening and assessment, developmental outcomes, developmental surveillance, hearing screening

## Abstract

**Background:**

Small infants face more developmental risks than their full-term peers, necessitating early intervention and long-term monitoring.

**Objectives:**

This study examined the longitudinal developmental and hearing outcomes of small infants attending a high-risk clinic in a South African low-income community setting.

**Method:**

A short-term longitudinal within-subject descriptive study design was employed, where 28 participants underwent hearing and developmental screenings and assessments at two follow-up appointments (T1 and T2), at 6- and 12-month corrected age. Developmental outcomes, such as communication, motor and daily living skills, were evaluated using developmental screening tools (Parents Evaluation of Developmental Status [PEDS]), hearing screening (ABR MB11) and developmental assessments (Vineland-3).

**Results:**

All participants underwent hearing screening, with four (14.3%) failing twice (T1 and T2) and being referred for diagnostic evaluation. Developmental screening at T1 identified concerns in communication, gross motor and social-emotional skills (28.5%). Concerns persisted across T1 and T2 in the PEDS tool, with fine motor skills emerging as a key issue at T2. Vineland-3 assessments showed improvement from T1 to T2; initial concerns in daily living (*M* = 104.12; standard deviation [s.d.] = 38.99) and motor skills (*M* = 88.82; s.d. = 45.26) were no longer present at T2, where all participants had age-appropriate developmental scores.

**Conclusion:**

The findings highlight the need for comprehensive, routine developmental monitoring and early intervention to address delays in small infants. Continued follow-up care and support from birth to 12 months corrected age can improve outcomes and caregiver developmental literacy.

**Contribution:**

This study provides valuable insights for caregivers, healthcare policymakers and early intervention professionals by emphasising the importance of early screening, continuous monitoring and caregiver education in optimising developmental outcomes for small infants.

## Introduction

Globally there is an increasing prevalence of small infants, of which 96% can be accounted for in low- and middle-income countries (LMICs) (Kassahun et al., 2019; Koenraads et al., 2017). Small infants are regarded as born weighing less than 2500 g at birth, including preterm and infants with low birth weight (LBW) (WHO, 2020b). These infants are at an increased risk of adverse developmental outcomes because of their immature central nervous systems and exposure to additional risk factors (Burnett et al., 2018). South Africa (SA) is considered an upper-middle-income country (UMIC); however, many communities within SA can be linked to LMIC status (Makgetla, 2020). The high incidence of preterm births in SA is directly associated with lower socio-economic circumstances (Buys & Gerber, 2021). Appropriate and timely developmental assessment and long-term monitoring of this small infant population are thus essential to identify potential developmental delays (Cheong et al., 2020; Spittle & Treyvaud, 2016).

A rise in the survival rate of small infants has been seen over the past several years because of advances in neonatal care and interventions (World Health Organization [WHO], 2020a). Research, however, shows that small infants are exposed to many risk factors and invasive procedures during their first 12 months, which can affect brain development (Moore et al., 2017; Pineda et al., 2018). An emphasis on long-term monitoring and improving the developmental outcomes of this at-risk population has been observed, focusing attention on follow-up care for at least 12 months (Cheong et al., 2020; Lipner & Huron, 2018). Reduced mortality rates have shifted the focus from survival to thriving, as the lower mortality rates in high-risk populations show higher developmental difficulties later in life (WHO et al., 2018). Long-term developmental monitoring faces limitations because of factors such as attrition in follow-up and constrained access to healthcare services in LMICs (Ramdin et al., 2022; Schoeman et al., 2017).

Apart from hospitalisation and procedures, small infants in LMICs also face a heightened risk of developmental delay, largely attributed to exposure to environmental risk factors. These factors include low maternal education and familial stress stemming from financial constraints (Black et al., 2017). Consequently, these infants encounter additional challenges in their development, as they often lack adequate stimulation and caregivers lack access to essential knowledge regarding developmental outcomes (Van Schalkwyk et al., 2020). Biological and environmental risks, such as prematurity, LBW and low maternal education among others, have a negative cumulative effect on infants and caregivers, highlighting the importance of understanding long-term developmental outcomes and monitoring (Bamford, 2019). This will assist in early identification, particularly during their first 1000 days (Bamford, 2019).

The first 1000 days of an infant’s life are a critical developmental period, particularly to mitigate the impact of early harmful actions, such as prolonged hospital stays, during this period (Richter et al., 2017). The effects of the harmful actions often manifest before 24 months corrected age, frequently emerging within the first 12 months (Hsu et al., 2018; Yaari et al., 2018). While essential for survival, extended hospitalisations can place stress on infants’ immature systems (Richter et al., 2017). The importance of a continuum of care the infants receive from birth through critical periods of development, including interventions at birth and during follow-up after hospitalisation, is highlighted (Smith et al., 2017).

To ensure optimal development, infants require ongoing medical and developmental monitoring post-discharge (Hsu et al., 2018; Linsell et al., 2018). Close monitoring of all developmental domains during this period is crucial, especially for small infants (Lipkin & Macias, 2020; Mareva & Holmes, 2019). This approach is consistent with developmental supportive care (DSC) and the nurturing care framework (NCF), which emphasises early, comprehensive monitoring following hospital discharge (WHO et al., 2018). Developmental delays often become evident between 6 and 12 months, coinciding with critical growth periods (Puthussery et al., 2018), emphasising the need for monitoring during this critical period (WHO et al., 2018). For instance, speech and language delays can affect other developmental domains, threatening overall development (McGowan & Vohr, 2019). Thorough monitoring during this period is therefore essential for prompt identification and addressing developmental concerns.

Communication delays are known to be one of the most prevalent childhood developmental delays, particularly in the small infant population, with late language emergence being one of the most common symptoms of developmental disability under the age of three (Fouché et al., 2019). Poor communication outcomes may influence other developmental domains and later scholastic performance (Lipkin & Macias, 2020). Previous studies have focused solely on specific developmental domains, such as the infants’ communication abilities (Zimmerman, 2018); it is, however, imperative that all developmental domains be monitored to provide a holistic perspective (Lipkin & Macias, 2020).

A study conducted in a South African low-income community considered late preterm infants’, born between 34 and 36 weeks of gestation, developmental outcomes every 3 months from 6 to 9 months corrected age, using the Bayley’s Scales of Infant and Toddler Development (BSID III), including all relevant developmental domains (Ramdin et al., 2018). This was one of the first longitudinal studies to report on the developmental outcomes of late preterm infants in SA. The study revealed that 7.1% of late preterm infants, between 34 and 36 weeks gestational age, had evidence of a developmental disability. This indicates the necessity of developmental screening and understanding developmental trajectories in this population, particularly in low-income communities, in SA (Ramdin et al., 2018). Moreover, the study’s focus was limited to the outcomes of late preterm infants assessed solely through the BSID III tool. This underscores the necessity of comprehensively examining the developmental outcomes of the entire continuum of the small infant population. A continuum of care involves integrating services and the appropriate professionals to deliver these services in resource-constrained settings in LMICs to ensure at-risk infants receive appropriate care from pregnancy right through childhood. It ensures the implementation of Kangaroo Mother Care (KMC) programmes and long-term developmental monitoring, which includes support, guidance, management and intervention (Schuler et al., 2023). Employing a broader range of screening and diagnostic tools to evaluate infants’ developmental trajectories can offer a more comprehensive understanding of early childhood development (ECD), encompassing various developmental domains (Enelamah et al., 2024; Schiariti et al., 2021).

Understanding small infants’ developmental trajectories can provide early interventionists and caregivers with important information concerning the developmental process (Schoeman et al., 2017). This information highlights the importance of routine monitoring of small infants’ development and encourages responsive hospital service delivery and relevant policy additions or standard operating procedures (SOPs) aligning with existing frameworks such as the DSC and NCF (WHO, 2020a; WHO et al., 2018). Therefore, the following research question was posed: What are the longitudinal developmental outcomes of small infants attending a South African high-risk follow-up clinic?

## Research methods and design

### Setting and participants

A high-risk follow-up clinic at a public tertiary hospital in a low-income community in South Africa was used as the setting in which data were gathered. Small infants who attended the high-risk follow-up clinic had previously been admitted to the neonatal intensive care unit (NICU), and down-referred to high-care and the KMC units before hospital discharge. The clinic offers services from various healthcare professionals, including audiologists, dieticians, doctors, nurses, occupational therapists and speech-language therapists. All of the healthcare professionals monitor the small infants, usually every 3 months at their follow-up appointments, up until 12 months corrected age. Participants in this study represented the caregiver-infant dyad, where the caregivers reported on the small infants’ development and observations were made on developmental outcomes, at two time points. The sample size of this study consisted of 28 caregiver-infant dyads, with small infants between the ages of 22 to 26 weeks and 50 to 54 weeks corrected age.

At the study’s first follow-up appointment (T1), participants were between the ages of 22 and 26 weeks (*M* = 23.9; standard deviation [s.d.] = 1.3), corrected age, where *M* and s.d. denote the mean and standard deviation, respectively. At the second follow-up appointment, 11 of 28 participants defaulted, resulting in a decreased sample of 17 participants. At T2, the infants were between the ages of 49.5 and 52 weeks (*M* = 50.4; s.d. = 0.1). Infants at the high-risk follow-up clinic are typically followed up until 12 months corrected age and discharged thereafter, if all stakeholders agree.

Non-probability purposive sampling was implemented to select participants who met the eligibility criteria: preterm, LBW infants with a birth weight of lower than 2500 g and a gestational age of less than 37 weeks at birth were classified as ‘small’ in this study. The participants represented a group of infants where no syndromes or congenital abnormalities were identified or diagnosed. Infants with identified or diagnosed syndromes or congenital abnormalities are expected to present with developmental delays; therefore, to determine the cumulative and direct impact of prematurity and LBW on an infant’s developmental outcomes, those with abnormalities were excluded from the study. Caregivers included in this study were all primary caregivers of the infants, including mothers, fathers, grandparents and older siblings.

### Materials and apparatus

The following assessment tools and caregiver questionnaires were used to obtain pertinent information concerning the small infants’ developmental profiles at both appointments. A combination of both screening and diagnostic tools was used in this study; all the tools were administered in English.

#### Parents Evaluation of Developmental Status tools

The Parents Evaluation of Developmental Status (PEDS) and PEDS: Developmental Milestones (PEDS:DM) are screening tools administered together, referred to as the PEDS tools, to provide comprehensive information on both the parents’ concerns and more specific information on developmental progress. Developmental domains included in the PEDS tools are as follows: fine motor, gross motor, social-emotional, self-help, cognitive, expressive and receptive language skills (Glascoe & Robertshaw, 2007). The screening tool relies on caregiver report, which is considered best practice in multilingual populations (Faruk et al., 2020). The PEDS tools consist of 16 questions concerning infants’ development in all relevant developmental domains.

The tools follow a specific algorithm for referral, which includes five paths, Paths A–E for the PEDS. If one or more milestones are not met on the PEDS:DM, the outcome of the screening results indicates a referral regardless of the PEDS result. The five paths are as follows:

- Path A – two or more predictive concerns in specific areas are present; namely, receptive language skills, school, social or self-help, which warrant a diagnostic referral to relevant healthcare professionals.
- Path B – one predictive concern present. Further screening is recommended.
- Path C – non-predictive concerns are present. Counselling should be provided to the caregivers.
- Path D – caregivers have difficulty communicating their concerns, possibly because of a language barrier.
- Path E – no concerns are present. Screening is considered a pass.

Paths B, C, D and E depend on the PEDS:DM result. This guides the healthcare professional on whether a pass result or further screening is indicated (Maleka et al., 2019).

The paper-based version of the PEDS tools was used to collect data regarding caregivers’ concerns about their infant’s development domains.

#### Vineland Adaptive Behaviour Scales, Third Edition (Vineland-3)

The Vineland-3 is a developmental diagnostic assessment tool used to measure personal and social skills needed for activities of daily living. The tool is suitable for various settings, including low-resourced LMICs (Sparrow et al., 2016). It was previously used in several local studies within SA (De Beer et al., 2020; Du Toit et al., 2021; Wrigglesworth et al., 2023). For this study, developmental assessment through caregiver report was obtained through means of a semi-structured interview using the comprehensive interview form, which included closed-ended questions. Domains and subdomains, including communication: receptive and expressive language skills; daily living skills: personal skills; socialisation: play and leisure and interpersonal relationships; as well as motor skills: gross and fine motor skills, were assessed (Sparrow et al., 2016).

The data obtained on the Vineland-3 indicated a delay when the adaptive behaviour composite (ABC) standard score, a combination of communication, daily living skills and socialisation, as well as the motor skills standard score, was more than one s.d. (15) below the mean of 100.

#### Automated Auditory Brainstem Response test MB 11 BERAphone

Small infants are at high risk for hearing impairments and can have further negative impacts on developmental outcomes, if not identified early (Kishino et al., 2021). An auditory brainstem response (ABR) was conducted to measure the neural activity of the auditory pathway. This test forms part of the standardised care provided upon each follow-up visit at the high-risk clinic. The ABR test can be used to identify hearing concerns in infants, both in the inner ear and subcortical auditory system (Gage & Baars, 2018). The screener was used to detect whether there was a possible hearing impairment present in the participants. A pass is considered when a response is detected and verified at 35 dB nHL (Kishino et al., 2021). The participants who failed the screening were referred for further diagnostic hearing testing and audiological management.

#### The Road to Health Booklet

The Road to Health Booklet (RTHB), which is provided to all caregivers upon their infants’ birth, was implemented by the South African Department of Health in October 2010 as a developmental surveillance tool to improve the service delivery provided to infants and young children (Van der Linde et al., 2015). The booklet aims to advise caregivers on their infant’s development and allows healthcare professionals to track and monitor an infants’ health, hearing test outcomes, growth and developmental trajectories at each follow-up opportunity. In this study, the booklet was used to obtain information such as the infants’ birth weight and gestational age, along with any additional information concerning birth, history and developmental status. Additionally, the booklet was used to keep track of the infants’ scheduled follow-up appointment dates.

### Data collection procedures

Caregiver-infant dyads were identified based on the sampling method, with assistance from the managing doctor at the follow-up clinic. No identifying information was included in the data collection and analysis process. Alphanumeric codes were assigned to ensure that no identifying information was used in this study. The identified participants were provided with the study information and invited to participate. Once caregivers agreed to participate, they provided written informed consent at the first data collection point (T1).

Infants in this study attended the high-risk follow-up clinic more frequently than on the two occasions on which data were collected (T1 and T2). Their first high-risk clinic appointment occurred 2 weeks after discharge from the KMC unit, with regular follow-up visits scheduled until they reached 12 months corrected age. This study collected data at two time points only: at 6 months corrected age (T1) and again at 12 months corrected age (T2). During these two study-specific appointments, additional procedures were conducted, including the administration of developmental screening and diagnostic tools and a hearing screening, which added approximately 30 min to the duration of their routine clinic visit.

At both T1 and T2, the diagnostic and screening tools (PEDS and Vineland-3) were administered through structured caregiver interviews. Hearing screening was also conducted at both time points. If a participant had not been screened at T1, the screening was completed at a subsequent follow-up clinic visit, and the results were documented in the infant’s clinic file. In cases where the infant failed the initial hearing screening, a retest was scheduled for the next available follow-up appointment.

Following the T1 appointment, caregivers were provided with a scheduled follow-up date for T2, approximately 6 months later. If any clinical concerns were identified by healthcare professionals at the clinic in the interim, caregivers were offered an earlier follow-up appointment. To support attendance, the researcher sent SMS reminders to participants at three intervals: 2 weeks before, 1 week before and 1 day before the scheduled follow-up.

All infants in the study received ongoing care from birth as part of a world-renowned skin-to-skin care programme during hospitalisation (Gooding et al., 2021). This programme forms part of routine care in the NICU, high-care and KMC units at the tertiary hospital, where caregivers roomed in with their infants. After discharge, the infants were routinely followed up at the high-risk clinic, where a multidisciplinary team, including audiologists, doctors, dieticians, nurses, occupational therapists, physiotherapists and speech therapists, conducted assessments and provided care. This interprofessional team collaborated to ensure that recommendations and interventions were holistic and tailored to the needs of both infants and caregivers.

When developmental or health concerns were identified during the study appointments at T1 or T2, the researcher made appropriate referrals to other members of the hospital’s interdisciplinary team. These referrals included audiologists, occupational therapists and doctors, ensuring that all infants received comprehensive and coordinated early intervention services.

### Data analysis

Descriptive statistics, one measure of location (*M*) and one measure of spread (s.d.), frequencies and percentages were used to collectively organise and summarise the raw data, based on the scores from all scoring sheets, in a meaningful manner (Pietersen & Maree, 2019).

Inferential statistics were employed to draw conclusions and generalise the results of the developmental outcomes of the small infants, whereby the statistics and parameters of the data collected were reported. Friedman’s two-way analysis ranks test was used to further analyse the data obtained from the Vineland-3. Statistical assumptions concerning the standard scores and *v*-scale scores obtained on the Vineland-3 domains and subdomains were made (Field, 2018).

Categorical variables were applied where the responses at the two points in time yielded binary data. McNemar’s test two-sided *p*-value, along with the test statistic denoted by a ‘*z*’, is reported in this study. McNemar’s test is a statistical test used to analyse paired categorical data (McNemar, 1947). It is commonly employed when comparing the proportions or frequencies of two related categorical variables collected from the same subjects or matched pairs. This was used to show the progression over the two data collection points, T1 and T2, concerning the outcomes obtained on the PEDS screening tools.

The non-parametric Wilcoxon-signed rank test (WSR) is used to test for differences between two related or dependent variables when data are non-normal. It’s the non-parametric equivalent to the parametric related-samples *t*-test (Field, 2018). The overall domains are reported in terms of WSR and whether a significant *p*-value, *p* < 0.05, was achieved when making comparisons between T1 and T2 on the Vineland-3.

### Ethical considerations

Ethical clearance to conduct this study was obtained in June 2022 from the Research Ethics Committee of the Faculty of Humanities, University of Pretoria, the Faculty of Health Sciences, University of Pretoria (reference number HUM016/0322) and the Ethics Committee at the Kalafong Hospital (reference number KPTH/June/2022).

## Results

Data were collected at two collection points, T1 and T2. At T1, the infant participants had a corrected mean age of 23.88 weeks (s.d. = 1.27) with a range between 22 and 26 weeks, and at T2, a mean corrected age of 50.38 weeks (s.d. = 0.09) with a range between 49.5 and 52 weeks (Table 1). The initial sample consisted of 28 participants (T1); however, at T2, only 60.7% (*n* = 17) participants attended their follow-up appointment, despite multiple reminders to prevent attrition. The sample was represented by both male and female infant participants, with different ratios at both T1 and T2; however, there were more male than female participants at both data collection points (Table 1). The sample included single births and multiple births, twins and triplets. The final sample of participants (*n* = 17) had a mean birth weight of 1781.88 g (s.d. = 635.04), indicating LBW and a mean gestational age of 32.24 weeks (s.d. = 3.6), indicating moderate preterm birth.

**TABLE 1:** Description of participants at T1 (*n* = 28) and T2 (*n* = 17).

Biographical information	T1		T2	
	*n*	s.d.	*n*	s.d.
**Gender**				
Male	15	-	10	-
Female	13	-	7	-
**Baby plurality**				
Singles	18	-	9	-
Twins	4 (2 sets)	-	2 (1 set)	-
Triplets	6 (2 sets)	-	6 (2 sets)	-
Mean corrected age (weeks)	23.88	1.27	50.38	0.09

s.d., standard deviation.

All 28 participants underwent a hearing screening, four participants (14.3%, *n* = 4/28) failed the hearing screening test twice, at T1 and T2, and were referred for a diagnostic hearing evaluation. The hearing screening occurred at T1 for the majority of the infants (82.14%, *n* = 23/28); however, some of the infants were not able to undergo the hearing screening at T1 because of external factors such as noise in the environment. The participants who were not screened at T1 were then screened at their subsequent follow-up appointment at the clinic and the results were recorded in their clinic file. Two of the four participants who failed the hearing screening presented with no developmental concerns across the PEDS tools and Vineland-3 domains (Table 2). One participant (*P3*, 3.57%, *n* = 1/17) who failed the hearing screening at T1 also presented with developmental concerns regarding language comprehension and expression, gross and fine motor skills, play and personal domains. At T2, however, no developmental concerns were identified regarding Participant Three (*P3)*. Participant Four (*P4*), with identified concerns in language comprehension and expression as well as gross and fine motor skills, defaulted on the follow-up appointment (T2). It was, however, indicated in the clinic file that the caregiver needed to be contacted to book further hearing and developmental assessment for the infant.

**TABLE 2:** Developmental outcomes of the participants who failed the hearing screening at T2 (*n* = 17).

Participant	HS	Language		Gross motor	Fine motor	Play	Personal
		Comprehension	Expression				
P1	Fail	-	-	-	-	-	-
P2	Fail	-	-	-	-	-	-
P3	Fail	^R^	^R^	^R^	^R^	^R^	^R^
P4	Fail	^R^	^R^	^R^	^R^	-	-
Frequency (*n*)	4	2	2	2	2	1	1

HS, Hearing screening.

^R^, Developmental domain refers to those identified with the PEDS tools or delays with the Vineland-3.

Referred outcomes and developmental delays were identified across the screening and assessment tools. At T1, four (14.3%, *n* = 4/28) participants who referred on the PEDS screening tools displayed delayed outcomes on the Vineland-3 across various domains, including communication and motor skills.

The data obtained from the PEDS screening tools at T1 indicated that eight (28.5%, *n* = 8/28) of the participants obtained a refer outcome in the developmental screening, with referred results in the domains of comprehension (10.7%; *n* = 3/28) and expressive language (10.7%; *n* = 3/28), social-emotional (10.7%; *n* = 3/28) and gross motor skills (7.1%; *n* = 2/28). In the PEDS tools, the communication domains, language comprehension and expression, along with social-emotional skills, were the domains with the highest concern at T1. Developmental domains that indicated age-appropriate screening outcomes at T1 concerning the PEDS tools were fine motor and self-help skills.

At T2, four (23.5%, *n* = 4/17) participants referred on the PEDS tools screening. The PEDS screening did not change significantly from T1 to T2 (*z* = 0.000; *p* = 1.000). Motor skills, specifically fine motor skills (23.5%; *n* = 4/17), was the domain with the poorest screening outcome at T2, resulting in referrals to physiotherapists or occupational therapists. At T2, one participant (5.9%; *n* = 1/17) had a refer outcome in the domains of language comprehension, expression and self-help skills. No concern was indicated for any participants regarding social-emotional skills at T2.

The diagnostic developmental assessment tool, Vineland-3, indicated three participants (10.7%, *n* = 3/28) scored at least one s.d. below the mean in all the relevant domains: communication, daily living skills, socialisation and motor skills, indicating an overall developmental delay at T1 (Table 3). It was found that daily living skills (*M* = 104.12; s.d. = 38.99) and motor skills (*M* = 88.82; s.d. = 45.26), similar to the motor skills concerns on the PEDS tools, were the domains with the poorest outcomes at T1. The communication domain (*M* = 123.24; s.d. = 22.12) had the strongest outcomes at T1, particularly the language expression domain where no delay was indicated. There was a significant difference between the outcomes from the PEDS screening tools, where communication domains presented the poorest outcomes. Similarly, there was a discrepancy in the outcomes of self-help and daily living skills. The PEDS tools indicated no concerns regarding self-help skills, while the Vineland-3 identified daily living skills as having the poorest outcomes.

**TABLE 3:** Comparison of delayed developmental outcomes according to diagnostic assessment tool Vineland-3, at T1 (*n* = 28) and T2 (*n* = 17).

Developmental domains and subdomains	T1				T2				WSR	*p*
	Mean	s.d.	*n* †	%	Mean	s.d.	*n* †	%		
**Developmental domain**										
Communication	123.24	22.12	4	23.5	133.53	6.97	0	0	−2.806	0.002*
Socialisation	112.65	23.90	3	17.6	137.29	3.57	0	0	−2.772	0.003*
Daily living skills	104.12	38.99	8	47.1	136.65	5.65	0	0	−2.579	0.008*
Motor skills	88.82	45.26	10	58.8	135.41	4.49	0	0	−2.790	0.003*
**Developmental subdomains**										
Language comprehension	17.06	7.65	4	23.5	21.18	2.72	0	0	−3.020	0.001*
Language expression	21.71	1.34	0	0.0	22.12	1.27	0	0	−1.364	0.214
Play and leisure	13.18	8.00	10	58.8	21.71	1.70	0	0	−2.917	0.002*
Interpersonal	21.82	1.43	0	0.0	23.71	0.47	0	0	−3.357	< 0.001*
Personal	15.76	8.53	8	47.1	22.76	1.64	0	0	−2.447	0.011*
Gross motor	14.76	7.60	9	53.0	22.71	1.40	0	0	−3.076	0.001*
Fine motor	12.53	9.70	10	58.8	23.18	1.13	0	0	−2.971	0.001*

s.d., standard deviation; WSR, Wilcoxon-signed rank test.

† , Frequency (*n*) of participants displaying developmental delay.

* , Significant *p*-value < 0.05.

At T2, all of the participants achieved standard scores above the mean of 100 in all domains, indicating age-appropriate development across all relevant domains on the Vineland-3. An overall significant improvement is thus observed in the developmental outcomes from T1 to T2 when using the Vineland-3, across developmental domains and subdomains (Table 3). The strongest domain at T2 was socialisation (*M* = 137.29; s.d. = 3.57), with communication being the domain with the lowest mean score (*M* = 133.53; s.d. = 6.97). The communication domain was, however, still within the age-appropriate range.

Improvement, indicating potential catch-up development, is shown in terms of the data values on the Vineland-3 (Table 3) from T1 to T2. This is further validated by the WSR values and statistically significant *p*-values achieved across the developmental domains from T1 to T2. This was evident in the motor skills (WSR = −2.790; *p* = 0.003) and socialisation (WSR = −2.772; *p* = 0.003) domains particularly. Motor skills remained a concern on the PEDS tools at T2.

## Discussion

This study explored the developmental outcomes of small infants attending a high-risk follow-up clinic at a tertiary hospital in a low-income community in South Africa. The tertiary hospital is world-renowned for its high-risk neonatal care practices, particularly the implementation of its skin-to-skin care programme as well as follow-up monitoring during repeated clinic visits, of this at-risk population (Gooding et al., 2021). The study’s findings underscore the benefit of developmental monitoring and the inclusion of caregivers as active care team members, supporting already existing frameworks, such as the DSC and the NCF (WHO, 2020b; WHO et al., 2018). Including caregivers as part of developmental monitoring improves developmental literacy, which leads to improved developmental outcomes because of increased awareness and support (Brown et al., 2020). Furthermore, the findings highlight the crucial impact of care provided from birth, such as skin-to-skin programmes, and during critical follow-up periods where repeated clinic visits occur (Brown et al., 2020; Dhage et al., 2023; Klutse et al., 2022).

The study identifies a prevalence of developmental delays in small infants, with delays present at T1 on Vineland-3 (10.7%; *n* = 3/28) across various domains. High referral rates were noticed on the PEDS tools. The screening tool demonstrates greater sensitivity than the diagnostic tool, identifying more concerns, particularly in the younger infants as seen at T1 in this study (28.5%, *n* = 8/28). This increased sensitivity may stem from the challenges in accurately assessing younger infants and the developmental literacy of caregivers at this stage (Lipkin & Macias, 2020). The PEDS tool’s question about expressive language at T1, ‘Does your baby “talk” or make sounds when holding a toy or seeing a pet?’ illustrates how the tool captures early signs of communication. Caregivers may have limited awareness of typical developmental milestones at this stage, affecting their responses and the perceived accuracy of their concerns for younger infants (Brown et al., 2020; Glascoe & Robertshaw, 2007; Wrigglesworth et al., 2023). In addition to the referrals on the screening tools and identified delays in the diagnostic assessment tools, a few participants displayed hearing concerns across the follow-up appointments (14.3%; *n* = 4). This highlights the need for ongoing developmental monitoring and surveillance beyond the neonatal period (Lipner & Huron, 2018). Early hearing concerns can have significant consequences for language development, cognitive skills and social-emotional functioning if not identified and addressed promptly (Yoshinaga-Itano et al., 2017). The Joint Committee on Infant Hearing (JCIH) recommends ongoing surveillance throughout early childhood, particularly for high-risk infants such as those born preterm, because of the increased likelihood of delayed-onset or progressive hearing loss (JCIH, 2019). A comprehensive approach is essential when supporting at-risk infants during their first critical years through coaching parents on appropriate developmental care and literacy (Chung et al., 2020; Ramdin et al., 2022).

Previous studies in LMICs have used the Bayley’s Scales of Infant and Toddler Development (BSID III) to assess the developmental outcomes of small infants (Ballot et al., 2017; Ramdin et al., 2018). Both studies reported concerns regarding motor and communication domain outcomes. These studies, however, focused on the assessment of extremely and very LBW, between 34 and 36 weeks gestational age, infants and not on the LBW and moderate preterm birth infants, as in this study. Another study performed by Liu et al. (2021) reported limited data on the predictive value of the BSID III when assessing the developmental outcomes of this vulnerable population (Liu et al., 2021). The study, however, demonstrated that infants who achieved subnormal cognitive scores at 6 months corrected age did not necessarily display the same outcomes at subsequent follow-up appointments; many had caught up and were within the normal range, aligning with the findings of this study.

Language and motor skills emerged as areas of concern in this study, with participants showing concerning outcomes in expressive and receptive language as well as gross and fine motor skills across the tools at T1. At T2, promising outcomes were noticed in daily living, social and communication skills domains on the Vineland-3. This aligns with existing literature that highlights the potential for positive development in small infants with appropriate support in these domains (Fouché et al., 2019; Pascal et al., 2018). The findings emphasise the importance of targeted interventions and support strategies, including aiding caregivers in developmental literacy as part of the continuum of care these at-risk infants receive, to build on these developmental strengths and improvements seen in developmental outcomes during follow-up (Brown et al., 2020; Ross et al., 2018).

The study incorporates hearing screening as a crucial assessment component, recognising the potential impact of hearing concerns on developmental outcomes (Yoshinaga-Itano et al., 2017). Four participants who failed the hearing screening displayed delays across multiple domains, emphasising the need for early identification and intervention for hearing-related issues in this population (Hirvonen et al., 2018). A study carried out by Thangavelu et al. (2023) demonstrated a referral rate of 3.8% in the small infant population (Thangavelu et al., 2023). A similar study reported a referral rate of 4.5% (Neumann et al., 2020). This study further highlighted the importance of the JCIH recommendations regarding universal newborn hearing screening (UNHS) protocols, which should follow the 1-3-6 guidelines to identify hearing concerns and provide intervention as early as possible (JCIH, 2019; Neumann et al., 2020). The 1-3-6 guidelines follow the principles of hearing screening before 1 month, a hearing evaluation before 3 months and early intervention before 6 months, should it be indicated. In addition to hearing screening, the JCIH recommends that other areas of sensory functioning, such as visual screening, be considered in this population (JCIH, 2019). Visual screening, along with the consideration of retinopathy of prematurity to prevent visual impairment and blindness in this population, may add additional value to long-term developmental screening processes (Lipkin & Macias, 2020). The impact that hearing and other sensation concerns could place on relevant developmental domains, particularly long-term outcomes, is thus recognised and should be a part of routine screenings added to the continuum of care received, from birth to 12 months corrected age at high-risk clinics in SA.

Repeated developmental assessments yield valuable insights into the developmental progress of small infants and may indicate potential maturation and catch-up growth processes, as well as improved developmental literacy seen in caregivers (WHO, 2020a). Specifically, the results from Vineland-3 assessments demonstrate an optimistic trend, showing an overall enhancement in developmental outcomes from the initial assessment to subsequent follow-up appointments. These findings are particularly evident in the significant results demonstrated in the motor skills (WSR = −2.790; *p* = 0.003) and socialisation (WSR = −2.772; *p* = 0.003) domains. This positive trajectory is an encouraging sign, suggesting that interventions supported developmental processes including maturation and catch-up growth (Lipkin & Macias, 2020; Liu et al., 2021). Maturation involves biological and cognitive growth and development that enhances abilities and skills over time, while catch-up growth is an accelerated development period following delayed growth (Cheong et al., 2020; Ferreira et al., 2020; Kim, 2022). Understanding the factors influencing both processes and considering variations in developmental outcomes during follow-up can help caregivers and healthcare providers better support small infants, aiding them in reaching their full potential (Black et al., 2017).

Contrasting results emerge when examining the results obtained from screening and assessment tools, particularly concerning the communication, self-help and daily living skills domains on the PEDS tools and Vineland-3, respectively. Tool sensitivity should be considered for possible differences seen across domains (Faruk et al., 2020; Van der Linde et al., 2015). The PEDS tools demonstrated age-appropriate development for self-help skills, whereas the daily living skills domain on the Vineland-3 had the poorest outcomes, second to motor skills at T1. Communication was a highlighted domain on the PEDS tools; however, the Vineland-3 indicated fewer concerns regarding communication. This inconsistency underscores the importance of adopting a holistic ECD perspective when evaluating the developmental trajectories of these infants (Lipner & Huron, 2018). Relying solely on a single screening or diagnostic assessment tool might not capture the complete picture of the small infants’ developmental outcomes. Using multiple tools provides a more comprehensive perspective, especially when concerns are consistent across different tools (Spittle & Treyvaud, 2016). A multidisciplinary team approach involving multiple professionals can also enhance the accuracy and scope of the assessment, such as including visual screenings and further exploring differences noticed between instruments used. Furthermore, research has shown that South African children, for example, often perform better in certain domains such as social-emotional, self-help and gross motor skills than global expectations (Wrigglesworth et al., 2023; Zhang et al., 2021). This suggests that multiple other factors should also be considered when interpreting assessment results.

The significance of a holistic ECD perspective, especially in the first 1000 days, is highlighted, as different tools may capture different dimensions of development (Ferreira et al., 2020). This multi-dimensional and multidisciplinary approach is crucial for understanding the challenges and strengths of small infants over time (Cheong et al., 2020). The implications of these divergent results call for further research to delve into the underlying reasons for the observed trends.

The study has implications for healthcare policy and practice, particularly in the context of low-income communities in SA. Albeit small, the findings highlight the importance of long-term developmental monitoring beyond the neonatal period as well as aiding in the developmental literacy of caregivers during the continuum of care. This adds additional value to existing models such as DSC and NCF, with the implication that they should be adopted consistently in high-risk settings (WHO et al., 2018). Integrating screening and diagnostic tools, including hearing and other sensory screening, into routine healthcare practices and targeted interventions, such as skin-to-skin programmes before discharge from the hospital setting, can contribute to improved outcomes for small infants (Van Schalkwyk et al., 2020). This is particularly true in the low-income community context where infants are not only at risk of adverse developmental outcomes because of being born prematurely with LBWs, but where other environmental factors also play a role (Bamford, 2019; Black et al., 2017).

This study has several limitations, including a 39.3% attrition rate at the second follow-up (T2), which may impact generalisability and introduce bias if those lost to follow-up differ from those retained (Ballot et al., 2017; Ramdin et al., 2018). Addressing barriers such as transport and socioeconomic challenges, potentially through mobile health (mHealth) technologies, may help reduce attrition in future studies (Knop et al., 2024). The small sample size limits statistical power, and inconsistencies between tools, especially in self-help or daily living domains, point to issues of tool sensitivity and cultural context (Faruk et al., 2020; Van der Linde et al., 2015; Wrigglesworth et al., 2023; Zhang et al., 2021). The short follow-up period further restricts insight into long-term developmental outcomes.

Future research should prioritise larger, multi-site studies across diverse South African regions to improve generalisability and statistical robustness. Broader use of developmental assessment tools is needed to fully capture the range of infant development (Faruk et al., 2020; Lipner & Huron, 2018), and discrepancies between tools such as the PEDS and Vineland-3 underscore the need for more sensitive and specific measures (Van der Linde et al., 2015). Longitudinal studies are essential to track the lasting impact of early interventions on developmental outcomes (WHO et al., 2018). Research should also explore the effectiveness of specific components of care, such as KMC, and examine the influence of environmental and socioeconomic factors, caregiver support and developmental literacy. Integrating routine developmental screening into healthcare systems and evaluating the feasibility of mHealth interventions may enhance care in low-resource settings (Knop et al., 2024). Finally, a greater focus on early sensory screening and intervention is recommended to address potential developmental delays promptly.

## Conclusion

This study provides insights into the developmental outcomes of small infants attending a high-risk follow-up clinic at a tertiary hospital in a low-income community in South Africa. Through a comprehensive and multi-dimensional assessment approach, the study identified concerns in developmental outcomes, particularly at 6 months corrected age, in areas such as communication and motor skills, underscoring the vulnerability to delays of this population. The findings emphasise the need for targeted interventions and support strategies to address specific developmental challenges faced in small infants, supporting a continuum of care from birth throughout childhood. They call for the integration of comprehensive screening programmes into a continuum of care and the implementation of early and continuous interventions, such as KMC and long-term follow-up. The study contributes valuable knowledge to the field of neonatal care and developmental surveillance, aligning with already existing frameworks (DSC and the NCF), particularly in low-income settings (WHO et al., 2018). It highlights the critical importance of early, multi-faceted, and sustained support for the developmental well-being of small infants. The findings of this study pave the way for future research and policy adaptations and development aimed at improving outcomes for this vulnerable population, ultimately shifting the focus from mere survival to thriving.
